# Depressive Symptoms and 6-Year KCCQ Health Status in Chronic Heart Failure: Findings From the Cognition.Matters-HF Cohort

**DOI:** 10.1016/j.jacadv.2026.103415

**Published:** 2026-10-31

**Authors:** Philipp Winkelmann, Stephanie Wissel, Vladimir Cejka, György Homola, Caroline Morbach, Roxanne Sell, Stefan Frantz, Alexandra Wuttke, Mirko Pham, Guido Stoll, Stefan Störk, Anna Frey, Jan Traub

**Affiliations:** a Department of Internal Medicine I, University Hospital Würzburg, Würzburg, Germany; b University Heart and Vascular Center Frankfurt, Frankfurt, Germany; c Comprehensive Heart Failure Center, Department of Clinical Research and Epidemiology, University and University Hospital Würzburg, Würzburg, Germany; d Department of Neuroradiology, University Hospital Würzburg, Würzburg, Germany; e Department of Psychiatry, Psychosomatics and Psychotherapy, University Hospital Würzburg, Würzburg, Germany; f Department of Psychology, University of Konstanz, Konstanz, Germany; g Department of Neurology, University Hospital Würzburg, Würzburg, Germany

**Keywords:** chronic heart failure, depression, disease-specific health status, Kansas City Cardiomyopathy Questionnaire, patient-reported outcomes, prospective cohort study

## Abstract

**Background:**

Long-term disease-specific health status trajectories in ambulatory chronic heart failure (HF) are incompletely characterized, and the contribution of depressive symptoms remains uncertain.

**Objectives:**

The objectives of the study was to characterize 6-year Kansas City Cardiomyopathy Questionnaire Overall Summary Score (KCCQ-OSS) trajectories and distinguish persistent and visit-specific depressive symptom associations with health status.

**Methods:**

In the prospective Cognition.Matters-HF cohort, 148 patients with HF underwent assessments at baseline and 6, 12, 36, and 69 months. Linear mixed-effects models estimated KCCQ-OSS trajectories and prespecified correlates. Patient Health Questionnaire-9 (PHQ-9) scores were decomposed into between-person and within-person components. Sensitivity analyses addressed missingness, and exploratory lagged analyses assessed whether recent PHQ-9 worsening predicted subsequent KCCQ deterioration or adverse clinical events.

**Results:**

KCCQ-OSS remained stable over 69 months (78.0-76.6; change: −1.4; 95% CI: −4.6 to 1.8; *P* for time = 0.065), despite substantial individual variation. Higher between-person PHQ-9 burden (β: −3.44 per point; 95% CI: −4.00 to −2.88; *P* < 0.001) and within-person PHQ-9 worsening (β: −1.80; 95% CI: −2.50 to −1.11; *P* < 0.001) were associated with lower concurrent KCCQ-OSS. Recent PHQ-9 worsening did not predict subsequent KCCQ-OSS, a ≥5-point KCCQ decline, or all-cause death/HF hospitalization. NYHA functional class and 6-min walking distance, but not N-terminal pro–B-type natriuretic peptide, left ventricular ejection fraction, or executive function, were independently associated with KCCQ-OSS.

**Conclusions:**

In ambulatory chronic HF, average KCCQ-based health status remained stable over 6 years despite substantial individual variation. Depressive symptoms were strongly associated with concurrent health status, but recent worsening did not predict subsequent KCCQ deterioration or adverse clinical events.

Chronic heart failure (HF) management increasingly extends beyond traditional cardiac markers to patient-centered assessment of health status, because symptoms, physical limitation, and quality of life are central to clinical decision-making and prognostically informative. The Kansas City Cardiomyopathy Questionnaire (KCCQ) is a validated HF-specific instrument that is now widely used in trials and clinical care.^1^ Yet long-term KCCQ trajectories in stable ambulatory chronic HF remain incompletely characterized. Most longitudinal studies have used shorter follow-up, acute HF cohorts, or generic health-related quality of life instruments, leaving uncertainty about which potentially modifiable factors are associated with long-term KCCQ-based health status.^2^

Depressive symptoms are common in HF and are consistently associated with worse patient-reported health status. In ambulatory patients with advanced HF, the REVIVAL study demonstrated a strong association between depression and lower KCCQ-OSS after accounting for other comorbidities, and a recent prospective study similarly linked longitudinal changes in depressive symptoms to changes in KCCQ over 1 year.^3^, ^4^ Thus, the general association between depressive symptoms and HF-specific health status is well established. Intervention trials targeting depression in HF demonstrated that mood symptoms can improve, yet effects on HF-specific health status are inconsistent.^5^, ^6^ Less well characterized is whether, over longer-term follow-up, the depression–health-status relationship reflects predominantly persistent differences between patients, visit-specific deviations within the same patient, or both. Separating these components may provide a more precise longitudinal characterization of this established relationship without implying causality or a specific management strategy.

Cognitive impairment, especially executive dysfunction, is also common in HF and clinically relevant for self-care, adherence, and outcomes. However, whether executive dysfunction is independently associated with disease-specific health status after accounting for depressive symptoms and clinical HF severity remains unclear.^7^

Therefore, using repeated assessments from the Cognition.Matters-HF cohort,^8^, ^9^ we investigated 6-year KCCQ-OSS trajectories and decomposed depressive symptom scores derived from the Patient Health Questionnaire-9 (PHQ-9) into between-person burden and within-person change. We then quantified the relative importance of these depressive symptom components compared with HF severity markers, functional capacity, and executive function. In additional temporally ordered analyses, we examined whether recent worsening in depressive symptoms predicted subsequent health-status deterioration or adverse clinical events.

## Methods

### Study design, population, and ethics

This study is a post hoc analysis of the prospective, single-center Cognition.Matters-HF cohort. The cohort design and methods have been reported previously.^8^, ^9^, ^10^ The study was conducted in accordance with the Declaration of Helsinki and approved by the local ethics committee of the University of Würzburg (#245/10). All participants provided written informed consent.

Between 2011 and 2014, adult patients with chronic HF of any etiology were recruited. HF had to be present for at least 1 year and no longer than 15 years according to the then-current European Society of Cardiology guidelines.^11^ Exclusion criteria included clinically apparent neurological or psychiatric disease, in particular clinically manifest depression or dementia, previous intracranial hemorrhage, clinical stroke, carotid artery stenosis >50%, and contraindications to study procedures. Depressive symptoms were nevertheless assessed as a continuous patient-reported measure at each study visit. Sex was recorded as biological sex/sex assigned at birth, as documented in the study records, and categorized as female or male. Gender identity was not systematically assessed in the original cohort protocol.

### Follow-up assessments and clinical measures

Study visits were scheduled at baseline and after 6, 12, 36, and 69 months. Assessments included clinical examination, assessment of NYHA functional class, laboratory testing, 6-min walking distance (6-MWD), transthoracic echocardiography, depressive symptoms, cognitive testing, and patient-reported health status. Trained study personnel performed all examinations according to standard operating procedures at the study center.

Transthoracic echocardiography was performed by trained and internally certified personnel using high-resolution ultrasound systems. Standard 2-dimensional images and Doppler recordings were acquired according to guideline recommendations.^8^, ^9^, ^10^ Left ventricular ejection fraction (LVEF) was determined from offline analyses. N-terminal pro–B-type natriuretic peptide (NT-proBNP) was measured as part of the clinical laboratory assessment and log-transformed for statistical analyses because of its skewed distribution.

### KCCQ health status and depressive symptoms

The 23-item KCCQ was administered at each study visit. The primary outcome was the KCCQ Overall Summary Score (KCCQ-OSS) derived from the 23-item KCCQ; it ranges from 0 to 100, with higher values indicating better disease-specific health status. Clinically meaningful changes were interpreted using established thresholds of 5, 10, and 20 points.^12^

Depressive symptoms were assessed using the PHQ-9.^13^ To distinguish sustained interindividual differences in depressive symptom burden from intraindividual change, PHQ-9 values were decomposed into between-person and within-person components. The between-person component was each participant’s mean PHQ-9 score across available visits. The within-person component was the deviation of each visit-specific PHQ-9 score from that participant’s mean. Thus, the model separately estimated associations of chronically higher depressive symptom burden and visit-specific worsening or improvement within the same participant.

### Executive function assessment

Cognitive testing was performed using a standardized neuropsychological test battery. For the present analysis, cognition was represented by a predefined executive function composite score. The composite was calculated from T-standardized measures of selectivity of attention (Test Battery of Attentional Performance^14^), working memory (Digit Span Forward and Block Tapping Span Forward tests^15^), and visual/verbal fluency (Regensburg Word Fluency Test^16^ and the Five-points test^17^). T-scores were standardized according to age-, sex-, and education-adjusted reference values, with a mean of 50 and SD of 10 in the reference population. Executive function was included as the predefined cognitive covariate in the primary models.

### Statistical analysis

Continuous variables are presented as median (quartiles), and categorical variables as counts (percentages). Participant flow and missing data were summarized descriptively for each scheduled study visit. Longitudinal trajectories of KCCQ-OSS and selected clinical, psychological, and cognitive variables were estimated using linear mixed-effects models that included all available observations. Time was modeled as a categorical fixed effect to estimate visit-specific marginal means and model-based changes from baseline to 69 months. Models included a participant-specific random intercept and an unstructured within-person covariance matrix to account for within-person correlation of repeated measurements. *P* values for longitudinal trajectories refer to the overall effect of time across study visits.

Covariates for the primary correlate model were selected a priori on the basis of clinical relevance and prior evidence rather than data-driven screening. The model included demographic variables, established markers of HF severity, functional capacity, depressive symptoms, and executive function as the predefined cognitive domain of interest. The primary correlate analysis used a linear mixed-effects model with KCCQ-OSS as the dependent variable. Fixed effects included time in years, the PHQ-9 between-person component, the PHQ-9 within-person component, sex, age, NYHA functional class, log-transformed NT-proBNP, LVEF, executive function, and 6-MWD. Predictor-by-time interaction terms were included to assess whether covariates were associated with annual change in KCCQ-OSS. Regression coefficients are reported with 95% CIs and *P* values. Main effects represent associations with KCCQ-OSS level, whereas interaction terms with time represent associations with annual change.

To examine temporal ordering, additional prospective analyses evaluated whether recent changes in depressive symptoms preceded subsequent deterioration in health status. For each eligible index visit, recent PHQ-9 change from the preceding visit was calculated and related to KCCQ-OSS at the subsequent visit. Gaussian generalized estimating equations (GEE) with patient-level clustering modeled subsequent KCCQ-OSS, adjusting for current KCCQ-OSS, current PHQ-9, and the transition interval to account for the unequal follow-up intervals. Logistic GEE models analogously evaluated subsequent clinically meaningful KCCQ-OSS deterioration of ≥5 points, with a ≥10-point decline examined as a sensitivity threshold. Additional models adjusted for age, sex, NYHA functional class, and log-transformed NT-proBNP.

To evaluate adverse clinical events, time-updated Cox models examined the first occurrence of all-cause death or HF hospitalization. Current PHQ-9 and recent PHQ-9 change were entered simultaneously so that the change term represented prognostic information beyond the current depressive symptom level. A clinically adjusted model additionally included age, sex, NYHA functional class, and log-transformed NT-proBNP. As a sensitivity analysis addressing potential overlap between somatic depressive and HF symptoms, the first 2 PHQ-9 items were analyzed as the PHQ-2 and decomposed into between-person and within-person components. These additional analyses were considered exploratory and used available complete observations.

Sex was included as a prespecified covariate in the primary correlate model. Because of the limited number of women in the cohort, sex-stratified analyses were not performed; the sex term was interpreted as an adjustment variable rather than as a formal test of sex-specific effects.

Missing observations were described for each scheduled visit and by variable. For the primary longitudinal trajectory analyses, unadjusted linear mixed-effects models used all available observed outcome data under a likelihood-based missing-at-random framework. For the primary correlate model, missing values in model variables were handled using multiple imputation by chained equations under a missing-at-random assumption. The imputation model included the outcome, all variables in the analytic model, study visit/time, and variables related to missingness or prognosis where available. To improve plausibility of missing-at-random assumption, lagged values of longitudinal variables from previous study visits were incorporated as auxiliary predictors. Sixty imputed data sets were generated, and estimates were pooled according to Rubin rules. The primary analysis included all 148 participants. A complete-case sensitivity analysis was performed in participants who attended the 69-month visit; remaining intermittent missing values in model variables were handled using the same imputation approach.

To assess robustness against departures from the missing-at-random assumption, a missing-not-at-random delta-adjustment sensitivity analysis was performed. Progressively negative offsets of 0 to −25 KCCQ-OSS points (0, −2, −5, −10, −15, −20 and −25) were applied to imputed missing KCCQ-OSS values to represent scenarios in which participants with missing patient-reported outcome data had worse health status than predicted under the missing-at-random assumption. The primary model was refitted across delta values, and the stability of key estimates was compared.

Because death during follow-up may represent informative dropout in long-term patient-reported outcome studies, a Bayesian joint longitudinal-survival model was performed as one of the principal sensitivity analyses specifically addressing death-related informative dropout. The purpose of this analysis was not to assess the prognostic utility of KCCQ-OSS, but to evaluate whether death-related informative dropout materially affected the estimated KCCQ-OSS trajectory. To maximize the sample size, the longitudinal submodel was intentionally parsimonious, describing KCCQ-OSS as a linear function of time with participant-specific random intercepts and slopes. The survival submodel described time to death, with participants alive or lost to follow-up censored at last known contact, and included an association parameter linking the underlying KCCQ-OSS process with mortality. Convergence was assessed using the potential scale reduction factor (potential scale reduction factor <1.100).

Analyses were structured hierarchically, with KCCQ-OSS defined as the single primary outcome. The longitudinal KCCQ-OSS trajectory and the primary correlate model were considered the main analyses. Sensitivity analyses, analyses of additional longitudinal variables, and predictor-by-time interaction terms were interpreted as supportive and exploratory. Therefore, no formal multiplicity correction was applied; emphasis was placed on effect sizes, 95% CI, and consistency across sensitivity analyses. Analyses were performed using R (version 4.5.1, R Foundation for Statistical Computing). Linear mixed-effects models were fitted using *nlme*, estimated marginal means were derived using *emmeans*, multiple imputation by chained equations was performed using *mice*, and the Bayesian joint longitudinal-survival sensitivity analysis was performed using *JMbayes2*. GEE were fitted using *geepack*, and time-updated Cox models were fitted using the survival package. A 2-sided *P* value <0.05 was considered statistically significant.

## Results

### Cohort characteristics and follow-up

A total of 148 patients with chronic HF were included in the longitudinal Cognition.Matters-HF cohort. Baseline characteristics are summarized in Table 1. Among patients with available baseline clinical, cognitive, and KCCQ data, the median age was 65 years (56-72 years), 16% were women, and ischemic HF was the most common etiology (66%). Most patients were in NYHA functional class II (60%). The median 6-MWD was 400 m (340-460 m), median NT-proBNP concentration was 672 pg/mL (237-1,677 pg/mL), and median LVEF was 44% (38%-48%). Baseline disease-specific health status was relatively preserved, with a median KCCQ overall summary score of 81 (68-92).

**Table 1:** Baseline Characteristics of the Cognition.Matters-HF Cohort (N = 148)

Age (y)	65 (56-72)
Female	24 (16)
Body mass index (kg/m^2^)	28 (26-32)
Heart failure parameters	
Time since diagnosis of heart failure (years)	4 (1-10)
Ischemic heart failure	96 (66)
NYHA functional class I	41 (28)
NYHA functional class II	88 (60)
NYHA functional class III	19 (13)
6-min walking distance (m)	400 (340-460)
N-terminal pro–B-type natriuretic peptide (pg/mL)	672 (237-1,677)
Left ventricular ejection fraction (%)	44 (38-48)
Heart failure with preserved ejection fraction	24 (17)
Heart failure with midrange ejection fraction	71 (48)
Heart failure with reduced ejection fraction	53 (36)
Left atrial volume index (mL/m^2^)	38 (29-52)
Left ventricular end-diastolic volume (mL)	127 (110-162)
E/e’ ratio	10 (7-16)
Medical history at baseline	
Coronary artery disease	100 (69)
Myocardial infarction	80 (55)
Atrial fibrillation	32 (22)
Cardiovascular risk factors	
Arterial hypertension	118 (80)
Diabetes mellitus	43 (29)
Hyperlipidemia	106 (72)
Smoking (ever)	89 (60)
Medication	
Angiotensin-converting enzyme inhibitor	87 (59)
Angiotensin II receptor blocker	48 (32)
Angiotensin receptor-neprilysin inhibitor	0 (0)
Beta-blocker	132 (89)
Mineralocorticoid receptor antagonist	55 (37)
Sodium-glucose cotransporter-2 inhibitor	0 (0)
Cardiac glycoside	21 (14)
Thiazide	23 (16)
Loop diuretic	81 (55)
Nitrates	3 (2)
Calcium antagonists	16 (11)
Amiodarone	6 (4)
Statins	101 (68)
Ezetimibe	9 (6)
Acetylsalicylic acid	80 (54)
Platelet inhibitor	21 (14)
Vitamin K antagonist	46 (31)
Oral anticoagulant	1 (1)
Insulins	14 (10)
Oral antidiabetics	35 (24)
Antidepressant	12 (8)
Allopurinol	33 (22)
Cognitive testing	
Intensity of attention (T score)	42 (37-47)
Visual/verbal memory (T score)	45 (40-52)
Executive functions (T score)	46 (42-50)
Selectivity of attention (T score)	48 (43-53)
Working memory (T score)	46 (40-52)
Visual/verbal fluency (T score)	45 (40-49)
Disease-specific health status assessment	
KCCQ Overall Summary Score	81 (68-92)
Depression	
PHQ-9 (Patient Health Questionnaire)	4 (2-7)

Values are median (quartiles) or n (%).

E/e′ = ratio of early transmitral flow velocity to early diastolic mitral annular velocity.

Participant flow and missing data across study visits are shown in Figure 1 and Table 2. Of the 148 patients recruited at baseline, 132 attended the 6-month visit, 124 attended the 12-month visit, 107 attended the 36-month visit, and 67 attended the 69-month visit. By 69 months, 29 patients had died and 52 had dropped out or did not respond. KCCQ-OSS was available in 138 patients at baseline, 127 at 6 months, 121 at 12 months, 101 at 36 months, and 60 at 69 months.

**Figure 1:**
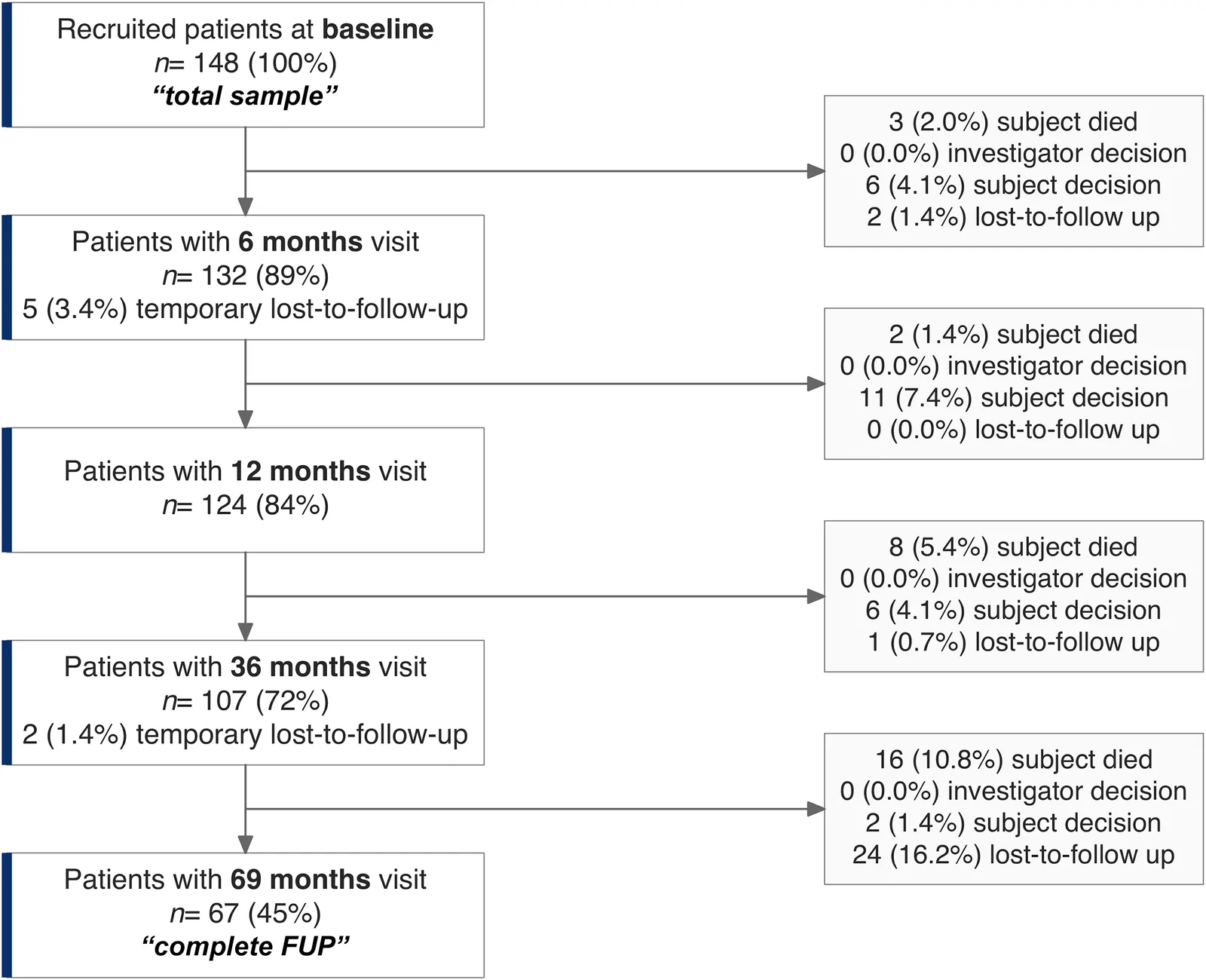
Cohort Flow and Follow-Up The figure summarizes participant flow in the prospective Cognition.Matters-Heart Failure cohort from baseline through 69 months. Among 148 enrolled patients with chronic heart failure, 132 attended the 6-month visit, 124 attended the 12-month visit, 107 attended the 36-month visit, and 67 attended the 69-month visit. By 69 months, 29 patients had died and 52 had dropped out or did not respond. Kansas City Cardiomyopathy Questionnaire Overall Summary Score availability at each visit is shown to illustrate longitudinal patient-reported outcome completeness.

**Table 2:** Participant Flow and Missing Data by Follow-Up Visit

	0 Months	6 Months	12 Months	36 Months	69 Months
Invited participants (n)	148	145	143	135	119
Participants assessed (n)	148	132	124	107	67
Deaths up to visit (n)	0	3	5	13	29
Dropouts/nonresponse (n)	0	13	19	28	52
Reason for missing follow-up data (n)					
Investigator decision	0	0	0	0	0
Participant decision	0	6	17	23	25
Lost to follow-up	0	2	2	3	27
Temporary loss to follow-up	0	5	0	2	0
Missing observations, n (%)					
KCCQ-OSS	10 (7)	21 (14)	27 (18)	47 (32)	88 (59)
PHQ-9	3 (2)	19 (13)	26 (18)	42 (28)	87 (59)
Age	1 (1)	16 (11)	25 (17)	44 (30)	88 (59)
Sex	0 (0)	0 (0)	0 (0)	0 (0)	0 (0)
NYHA functional class	0 (0)	16 (11)	25 (17)	41 (28)	82 (55)
NT-proBNP	6 (4)	24 (16)	30 (20)	54 (36)	87 (59)
Executive function	2 (1)	148 (100)^a^	26 (18)	50 (34)	98 (66)
Left ventricular ejection fraction	0 (0)	148 (100)^a^	26 (18)	42 (28)	83 (56)
6-min walking distance	8 (5)	24 (16)	28 (19)	49 (33)	91 (61)

The table summarizes scheduled visits, participant attendance, deaths, dropouts or nonresponse, and available observations for longitudinal outcomes and covariates.

KCCQ-OSS = Kansas City Cardiomyopathy Questionnaire Overall Summary Score; NT-proBNP = N-terminal pro–B-type natriuretic peptide; PHQ-9 = Patient Health Questionnaire-9.

a According to the study’s design, the assessment of both executive function and left ventricular ejection fraction was not conducted at the 6-month visit.

### Six-year trajectories of KCCQ-OSS and clinical markers

Model-based longitudinal trajectories are shown in Table 3 and Figure 2A. At the population level, mean KCCQ-OSS remained stable over 69 months. Estimated marginal means were 78.0 points at baseline, 79.1 at 6 months, 79.4 at 12 months, 76.1 at 36 months, and 76.6 at 69 months. The model-estimated change from baseline to 69 months was −1.4 points (95% CI: −4.6 to 1.8; *P* for time = 0.065).

**Table 3:** 6-Year Health Status and Clinical Trajectories

Outcome	Baseline EMM (95% CI)	6-Month EMM (95% CI)	12-Month EMM (95% CI)	36-Month EMM (95% CI)	69-Month EMM (95% CI)	Model-Estimated Change From Baseline to 69 Months (95% CI)	*P* Value	Observations, n
KCCQ-OSS	78.0 (75.1-80.9)	79.1 (75.9-82.3)	79.4 (76.4-82.5)	76.1 (72.5-79.7)	76.6 (72.8-80.3)	−1.4 (−4.6 to 1.8)	0.065	547
PHQ-9	5.1 (4.4-5.8)	5.1 (4.3-5.8)	4.9 (4.2-5.6)	5.8 (4.9-6.6)	5.8 (5.0-6.7)	0.7 (−0.1 to 1.5)	0.087	563
NYHA functional class	1.9 (1.8-2.0)	1.8 (1.7-1.9)	1.8 (1.7-1.9)	1.9 (1.8-2.1)	2.2 (2.0-2.4)	0.3 (0.1-0.5)	**0.001**	576
log(NT-proBNP)	6.4 (6.2-6.6)	6.3 (6.1-6.5)	6.3 (6.1-6.5)	6.3 (6.1-6.6)	6.4 (6.2-6.7)	0.0 (−0.2 to 0.2)	**0.033**	539
Executive function	46.2 (45.3-47.1)	–	45.9 (44.9-46.9)	46.8 (45.7-47.8)	46.4 (45.0-47.7)	0.2 (−0.8 to 1.1)	0.103	416
LVEF (%)	42.6 (41.2-43.9)	–	43.0 (41.5-44.6)	43.1 (41.2-45.1)	42.5 (40.5-44.5)	−0.1 (−2.1 to 2.0)	0.755	441
6-MWD (m)	389 (373-406)	392 (375-409)	394 (376-411)	385 (366-405)	387 (364-409)	−3 (−17 to 12)	0.443	540

Estimated marginal means with 95% CIs were derived from linear mixed-effects models using all available observations. Changes between baseline and 69 months are presented as model-based estimates. *P* values refer to the overall effect of time across all study visits. **Bolded** values indicates *P* Value < 0.05.

6-MWD = 6-min walking distance; EMM = estimated marginal mean; LVEF = left ventricular ejection fraction; other abbreviations as in Table 2.

**Figure 2:**
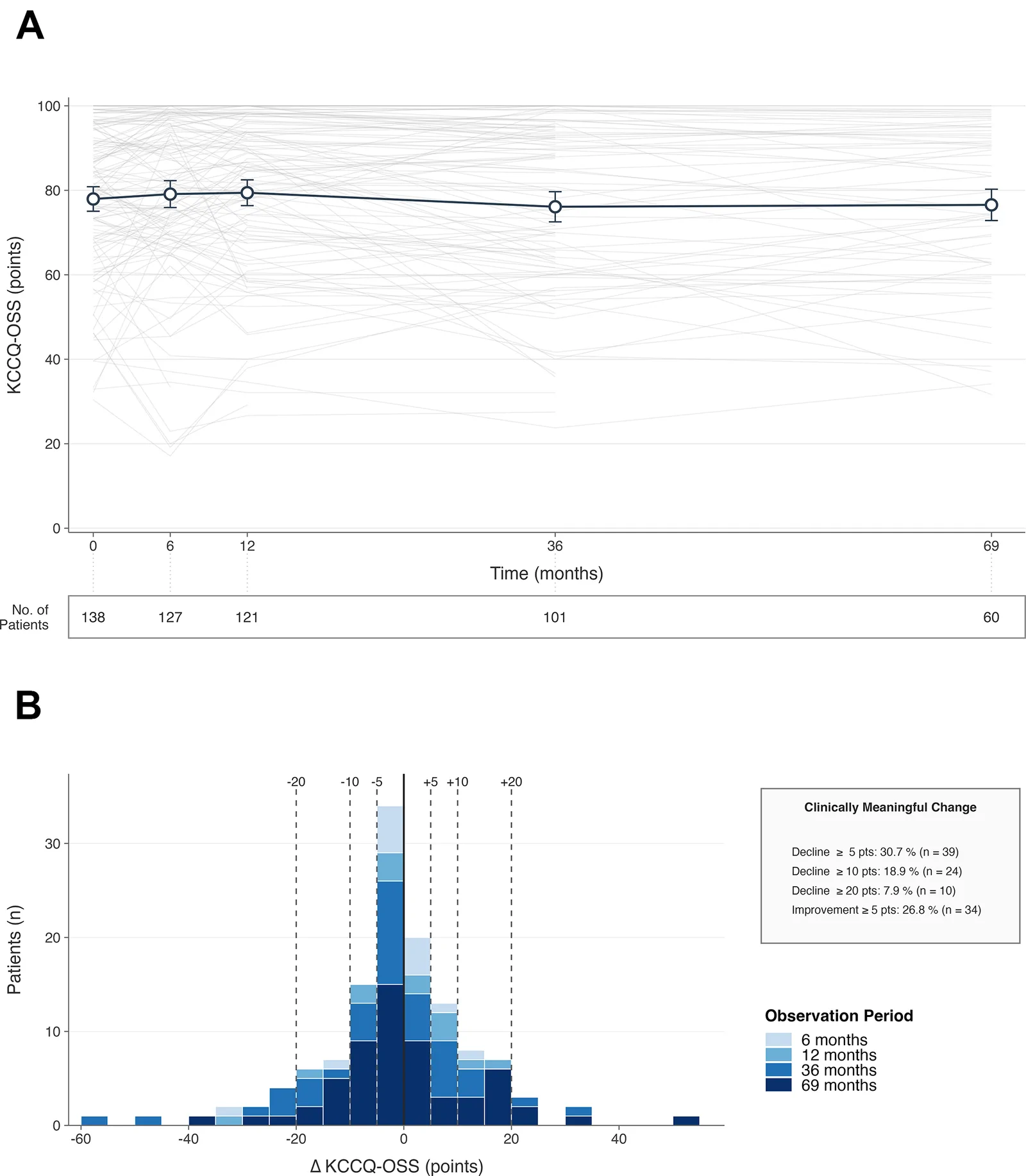
Longitudinal Kansas City Cardiomyopathy Questionnaire Overall Summary Score Trajectory and Individual Change Distribution (A) Individual observed KCCQ-OSS trajectories are shown in gray. Superimposed points and error bars represent visit-specific estimated marginal means and 95% CIs derived directly from the linear mixed-effects model with study visit modeled as a categorical fixed effect. No data-level smoothing function was applied. Numbers below the x-axis indicate the number of available KCCQ-OSS observations at each visit. (B) Distribution of individual change in KCCQ-OSS from baseline to each follow-up visit, with clinically meaningful change thresholds indicated. KCCQ-OSS = Kansas City Cardiomyopathy Questionnaire Overall Summary Score.

In contrast, NYHA functional class worsened modestly over time, increasing from 1.9 at baseline to 2.2 at 69 months (model-estimated change, 0.3 classes; 95% CI: 0.1-0.5; *P* = 0.001). PHQ-9 scores showed a nonsignificant increase from 5.1 to 5.8 points (change, 0.7; 95% CI: −0.1 to 1.5; *P* = 0.087). LVEF, executive function, and 6-MWD showed no relevant long-term change. Log-transformed NT-proBNP showed a statistically significant overall time effect (*P* = 0.033), but no relevant change between baseline and 69 months.

Despite this stable population-average trajectory, individual KCCQ-OSS changes were heterogeneous (Figure 2B). Among patients with available change scores, 30.7% had a decline of at least 5 points, 18.9% a decline of at least 10 points, and 7.9% a decline of at least 20 points, whereas 26.8% showed an improvement of at least 5 points.

### Correlates of KCCQ-OSS level and change

The primary mixed-effects model included all 148 patients and 547 KCCQ-OSS observations with missing model variables handled by multiple imputation under the missing-at-random assumption (Table 4). The intraclass correlation coefficient was 0.44, indicating substantial between-patient variability in KCCQ-OSS level.

**Table 4:** Correlates of KCCQ Overall Summary Score

Predictor	Effect on KCCQ-OSS Level, β (95% CI)	*P* Value	Effect on Annual Change in KCCQ-OSS, β Time × Predictor (95% CI)	*P* Value	Patients (n)/Observations (n)	ICC/Variance Components
PHQ-9 between-person effect^a^	**−3.44 (−4.00 to −2.88)**	**<0.001**	0.09 (−0.13 to 0.30)	0.435	148/547	ICC = 0.44 (σ^2^_u0 = 49.02; σ^2^_ε = 63.28)
PHQ-9 within-person effect^b^	**−1.80 (−2.50 to −1.11)**	**<0.001**	0.10 (−0.26 to 0.46)	0.596		
Sex	0.05 (−5.60 to 5.70)	0.987	0.58 (−1.80 to 2.96)	0.631		
NYHA functional class	**−4.72 (−7.51 to −1.94)**	**0.001**	−0.10 (−1.25 to 1.05)	0.861		
log(NT-proBNP)	−0.21 (−1.98 to 1.57)	0.819	−0.21 (−0.86 to 0.44)	0.524		
Executive function	−0.30 (−0.69 to 0.10)	0.139	0.02 (−0.12 to 0.16)	0.780		
LVEF	0.16 (−0.07 to 0.38)	0.177	−0.03 (−0.13 to 0.07)	0.534		
Age	0.06 (−0.21 to 0.33)	0.639	−0.02 (−0.12 to 0.08)	0.672		
6-min walking distance	**0.04 (0.01**-**0.07)**	**0.004**	−0.00 (−0.01 to 0.01)	0.436		

Results are from the primary linear mixed-effects model evaluating correlates of KCCQ-OSS under the missing-at-random assumption using multiple imputation. Estimates are regression coefficients with 95% CIs. Main effects represent associations with KCCQ-OSS level; time × predictor interactions represent associations with annual change.

ICC = intraclass correlation coefficient; other abbreviations as in Tables 2 and 3.

a PHQ-9 between-person effect represents each participant’s mean PHQ-9 score across available visits.

b PHQ-9 within-person effect represents the visit-specific deviation from each participant’s mean PHQ-9 score.

PHQ-9 showed the strongest association with KCCQ-OSS. Higher between-person PHQ-9 burden was associated with markedly lower KCCQ-OSS (β: −3.44 per PHQ-9 point; 95% CI; −4.00 to −2.88; *P* < 0.001). In addition, a visit-specific within-person increase in PHQ-9 relative to a participant’s own mean was independently associated with lower KCCQ-OSS (β: −1.80; 95% CI: −2.50 to −1.11; *P* < 0.001). Thus, both sustained depressive symptom burden and contemporaneous within-person worsening were associated with worse disease-specific health status.

Among clinical covariates, higher NYHA functional class was associated with lower KCCQ-OSS (β: −4.72; 95% CI: −7.51 to −1.94; *P* = 0.001), whereas higher 6-MWD was associated with higher KCCQ-OSS (β: 0.04 per meter; 95% CI: 0.01-0.07; *P* = 0.004). Sex, age, log-transformed NT-proBNP, LVEF, and executive function were not independently associated with KCCQ-OSS level.

No predictor showed a robust association with annual change in KCCQ-OSS. In particular, the interaction terms for time with PHQ-9 between-person burden (β: 0.09; 95% CI: −0.13 to 0.30; *P* = 0.435) and for time with PHQ-9 within-person change (β: 0.10; 95% CI: −0.26 to 0.46; *P* = 0.596) were not significant. The association between depressive symptoms and KCCQ-OSS was therefore reflected primarily in health-status level rather than progressive divergence of trajectories over time. The adjusted model-based association between within-person PHQ-9 deviations and KCCQ-OSS over time is illustrated in Figure 4.

**Figure 4:**
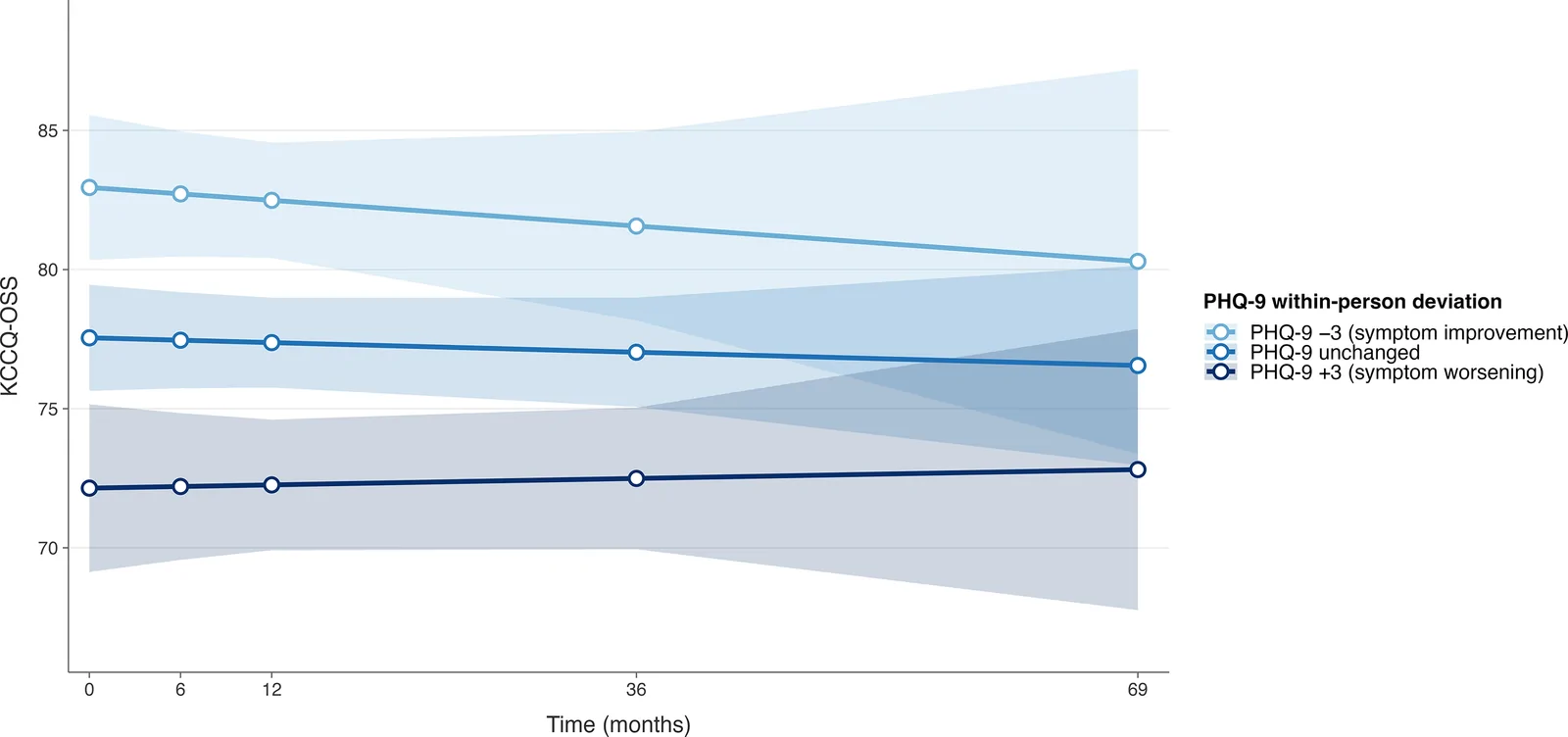
Depressive Symptoms and Kansas City Cardiomyopathy Questionnaire Overall Summary Score Predicted KCCQ-OSS trajectories are shown for within-person PHQ-9 deviations of −3, 0, and +3 points from each participant’s individual mean PHQ-9 score. Estimates were derived from the primary mixed-effects model and adjusted for age, sex, NYHA functional class, log(N-terminal pro–B-type natriuretic peptide), left ventricular ejection fraction, executive function, 6-min walking distance, and PHQ-9 between-person mean. Shaded areas indicate 95% CIs. Within-person worsening in depressive symptoms was associated with lower health status across follow-up. Abbreviations as in Figures 2 and 3.

### Principal sensitivity analyses: missing data and informative dropout

Results were consistent in the complete-follow-up sensitivity analysis restricted to 67 participants with 266 observations (Table 5). Higher PHQ-9 between-person burden remained strongly associated with lower KCCQ-OSS (β: −4.04; 95% CI: −4.76 to −3.33; *P* < 0.001), as did within-person PHQ-9 worsening (β: −2.02; 95% CI: −2.77 to −1.27; *P* < 0.001). Higher 6-MWD also remained associated with higher KCCQ-OSS (β: 0.04; 95% CI: 0.01-0.07; *P* = 0.010). NYHA functional class showed a borderline association with KCCQ-OSS level (β: −3.49; 95% CI: −6.99 to 0.01; *P* = 0.051). No predictor showed a significant association with annual KCCQ-OSS change.

**Table 5:** Complete-Follow-Up Sensitivity Analysis of KCCQ Correlates

Predictor	Effect on KCCQ-OSS Level, β (95% CI)	*P* Value	Effect on Annual Change in KCCQ-OSS, β time × predictor (95% CI)	*P* Value	Patients (n)/Observations (n)	ICC/Variance Components
PHQ-9 between-person effect^a^	**−4.04 (−4.76 to −3.33)**	**<0.001**	0.09 (−0.12 to 0.29)	0.398	67/266	ICC = 0.41 (σ^2^_u0 = 38.99; σ^2^_ε = 55.72)
PHQ-9 within-person effect^b^	**−2.02 (−2.77 to −1.27)**	**<0.001**	0.14 (−0.13 to 0.42)	0.312		
Sex	3.93 (−5.72 to 13.58)	0.425	−0.02 (−2.48 to 2.44)	0.986		
NYHA functional class	−3.49 (−6.99 to 0.01)	0.051	−0.08 (−1.08 to 0.91)	0.870		
log(NT-proBNP)	−0.83 (−3.34 to 1.68)	0.515	0.04 (−0.59 to 0.67)	0.896		
Executive function	0.02 (−0.38 to 0.42)	0.928	−0.00 (−0.11 to 0.10)	0.926		
LVEF	0.00 (−0.30 to 0.30)	0.993	−0.01 (−0.10 to 0.08)	0.804		
Age	0.07 (−0.22 to 0.36)	0.644	−0.06 (−0.14 to 0.01)	0.114		
6-min walking distance	**0.04 (0.01** to **0.07)**	**0.010**	−0.00 (−0.01 to 0.00)	0.320		

Results are restricted to participants with 69-mo follow-up and use the same linear mixed-effects model specification as the primary analysis. Estimates are regression coefficients with 95% CIs. Main effects represent associations with KCCQ-OSS level; time × predictor interactions represent associations with annual change.

Abbreviations as in Table 2, Table 3, Table 4.

a PHQ-9 between-person effect represents each participant’s mean PHQ-9 score across available visits.

b PHQ-9 within-person effect represents the visit-specific deviation from each participant’s mean PHQ-9 score.

The missing not at random (MNAR) delta-adjustment sensitivity analysis is shown in Figure 3. Applying progressively negative offsets to imputed missing KCCQ-OSS values did not materially alter the main estimates. In particular, the associations of PHQ-9 between-person burden, PHQ-9 within-person change, NYHA functional class, and 6-MWD with KCCQ-OSS remained stable across the examined delta values.

**Figure 3:**
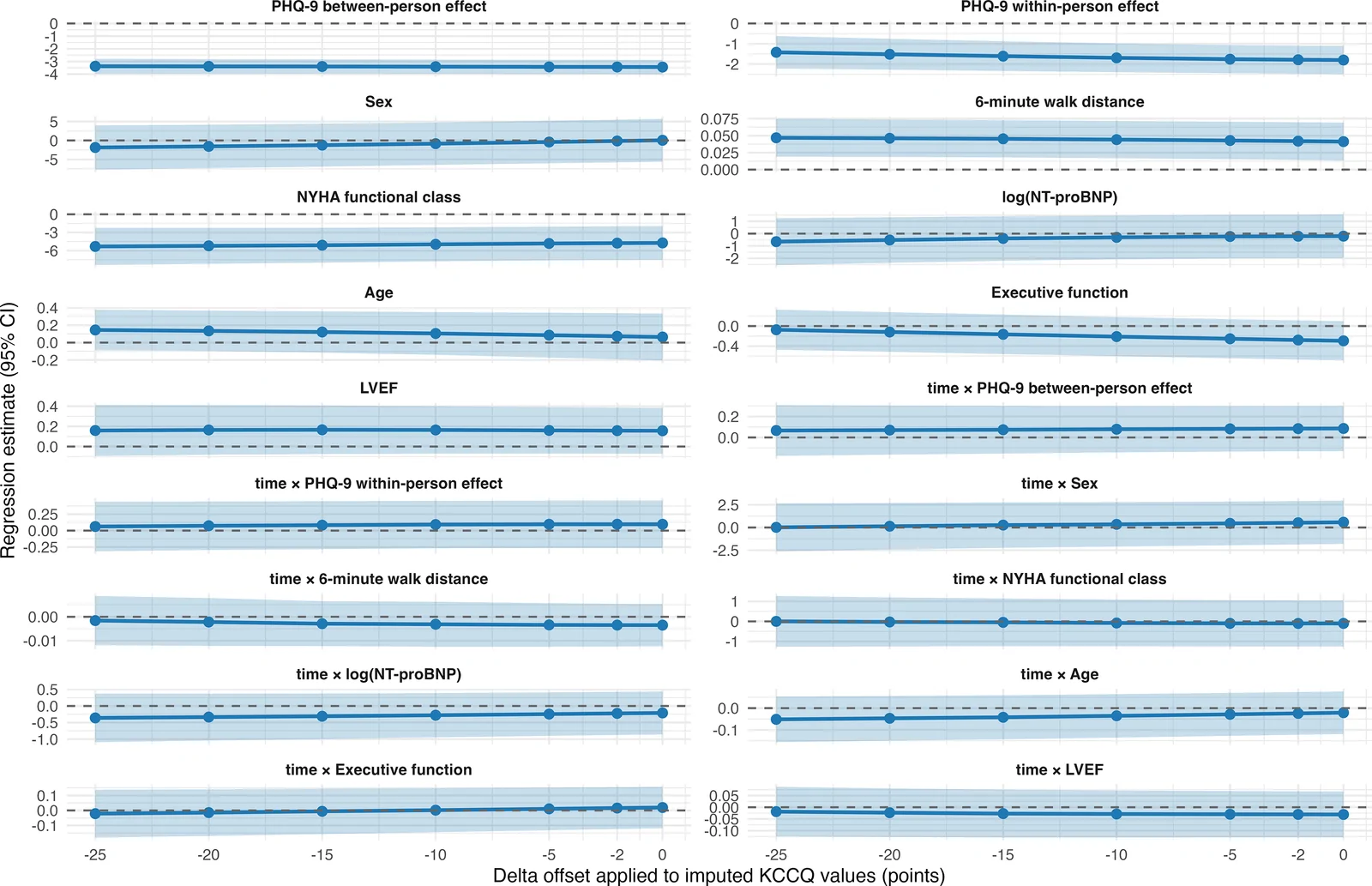
Missing-Data Sensitivity Analysis This figure shows the missing-not-at-random delta-adjustment sensitivity analysis for the primary mixed-effects model. Progressively negative offsets were applied to imputed missing KCCQ-Overall Summary Score values to examine whether worse-than-predicted health status among participants with missing data altered the main findings. The associations of PHQ-9 between-person burden, PHQ-9 within-person change, NYHA functional class, and 6-min walking distance with KCCQ-Overall Summary Score remained stable across the examined delta values. KCCQ = Kansas City Cardiomyopathy Questionnaire; LVEF = left ventricular ejection fraction; NT-proBNP = N-terminal pro–B-type natriuretic peptide; PHQ-9 = Patient Health Questionnaire-9.

As a principal sensitivity analysis specifically addressing death-related informative dropout, a joint longitudinal-survival model evaluated whether death during follow-up influenced the estimated KCCQ-OSS trajectory (Table 6). In this model, the annual KCCQ-OSS change was −0.44 points (95% credible interval: −1.25 to 0.38), and the association parameter linking KCCQ-OSS to survival was small (−0.013; 95% credible interval: −0.040 to 0.014). Thus, the joint model did not provide evidence that death-related informative dropout materially biased the estimated KCCQ-OSS trajectory.

**Table 6:** Joint Model Sensitivity Analysis for KCCQ-OSS

	Estimate	95% CrI	Rhat
Longitudinal Submodel			
Intercept	78.84	76.03-81.67	1.001
Time (per year)	−0.44	−1.25-0.38	1.000
Residual SD (σ)	8.03	7.33-8.78	1.003
Random effects			
SD of random intercepts	16.69	-	-
SD of random slopes (time)	2.70	-	-
Correlation between intercept and slope	−0.14	-	-
Survival submodel			
Association parameter (KCCQ-OSS)	−0.013	−0.040-0.014	1.004

Results are from a Bayesian joint longitudinal-survival model used to assess potential informative dropout due to death. The longitudinal submodel describes KCCQ-OSS trajectories over time, whereas the survival submodel evaluates the association between the underlying KCCQ-OSS process and mortality. Estimates are presented with 95% credible intervals.

CrI = credible interval; Rhat = potential scale reduction factor; other abbreviation as in Table 2.

In an additional sensitivity analysis, we restricted depressive symptom assessment to the PHQ-2, which captures the 2 core affective symptoms of depression—anhedonia and depressed mood—while excluding the somatic PHQ-9 items such as fatigue, sleep disturbance, appetite change, and psychomotor symptoms that may overlap with manifestations of HF. This analysis was intended to assess whether the strong contemporaneous PHQ-9–KCCQ association might largely reflect shared somatic symptom content. Both sustained between-person PHQ-2 burden and visit-specific within-person PHQ-2 worsening remained strongly associated with lower KCCQ-OSS. The within-person association persisted after adjustment for age, sex, NYHA functional class, and NT-proBNP (β: −4.02; 95% CI: −5.08 to −2.96; *P* < 0.001), indicating that the contemporaneous association was not solely explained by overlap between somatic depressive symptoms and HF symptom burden.

Taken together, the complete-follow-up, MNAR delta-adjustment, and joint longitudinal-survival analyses provided concordant evidence supporting the robustness of the main findings.

### Prospective associations of depressive symptom change

To examine temporal ordering, we evaluated whether recent worsening in depressive symptoms predicted subsequent deterioration in health status (Table 7). Across 261 transitions in 114 participants, recent PHQ-9 change was not associated with KCCQ-OSS at the subsequent visit after adjustment for current KCCQ-OSS, current PHQ-9, and transition interval (β: 0.24 per 1-point PHQ-9 increase; 95% CI: −0.37 to 0.86; *P* = 0.438). Results remained unchanged after additional adjustment for age, sex, NYHA functional class, and log-transformed NT-proBNP (β: 0.16; 95% CI: −0.46 to 0.78; *P* = 0.605).

**Table 7:** Prospective Associations of Recent PHQ-9 Change With Subsequent Outcomes

Subsequent Outcome	Model	Participants/Observations	Events	Effect of recent PHQ-9 Worsening (95% CI)	*P* Value
KCCQ-OSS at next visit	Primary	114/261 transitions	—	β = 0.24 (−0.37 to 0.86)	0.438
KCCQ-OSS at next visit	Clinically adjusted	111/245 transitions	—	β = 0.16 (−0.46 to 0.78)	0.605
KCCQ-OSS decline ≥5 points	Primary	114/261 transitions	60	OR = 0.92 (0.82-1.02)	0.117
KCCQ-OSS decline ≥5 points	Clinically adjusted	111/245 transitions	56	OR = 0.95 (0.84-1.07)	0.374
KCCQ-OSS decline ≥10 points	Sensitivity	114/261 transitions	24	OR = 0.87 (0.73-1.03)	0.103
Death or HF hospitalization	Primary time-updated Cox	131/323 risk intervals	45	HR = 1.05 (0.94-1.17)	0.401
Death or HF hospitalization	Clinically adjusted	129/298 risk intervals	45	HR = 1.01 (0.89-1.15)	0.872

Recent PHQ-9 change was defined as the current PHQ-9 score minus the score at the preceding study visit; positive values indicate worsening depressive symptoms. For continuous and binary KCCQ outcomes, primary models adjusted for current KCCQ-OSS, current PHQ-9, and transition interval. Clinically adjusted models additionally included age, sex, NYHA functional class, and log-transformed NT-proBNP. Time-updated cox models included current PHQ-9 and recent PHQ-9 change simultaneously; the clinically adjusted model additionally included age, sex, NYHA functional class, and Log-transformed NT-proBNP. Effect estimates are reported per 1-point increase in recent PHQ-9.

HF = heart failure; other abbreviations as in Table 2.

Similarly, recent PHQ-9 worsening was not associated with a subsequent clinically meaningful KCCQ-OSS decline of ≥5 points (60 events; OR: 0.92; 95% CI: 0.82-1.02; *P* = 0.117), including after additional clinical adjustment (OR: 0.95; 95% CI: 0.84-1.07; *P* = 0.374). Results were consistent using a ≥10-point deterioration threshold (24 events; OR: 0.87; 95% CI: 0.73-1.03; *P* = 0.103).

In time-updated Cox analyses comprising 323 risk intervals in 131 participants and 45 subsequent events, recent PHQ-9 change was not associated with subsequent all-cause death or HF hospitalization after accounting for the current PHQ-9 level (HR: 1.05; 95% CI: 0.94-1.17; *P* = 0.401). Additional adjustment for age, sex, NYHA functional class, and log-transformed NT-proBNP yielded a similar result (HR: 1.01; 95% CI: 0.89-1.15; *P* = 0.872).

## Discussion

In this longitudinal cohort of ambulatory patients with chronic HF, at the population level, KCCQ-OSS remained largely stable over 6 years despite modest worsening in NYHA functional class (Central Illustration). Importantly, this stable average trajectory masked substantial individual variation, with clinically meaningful deterioration in a considerable proportion of participants and improvement in others. The dominant correlates of worse KCCQ-OSS were depressive symptoms, captured both as sustained between-person burden and as visit-specific within-person worsening. The additional prospective analyses further refine this interpretation: although depressive symptoms were strongly associated with concurrent KCCQ-OSS, recent worsening in depressive symptoms did not predict subsequent health-status deterioration or adverse clinical events.

**Central Illustration:**
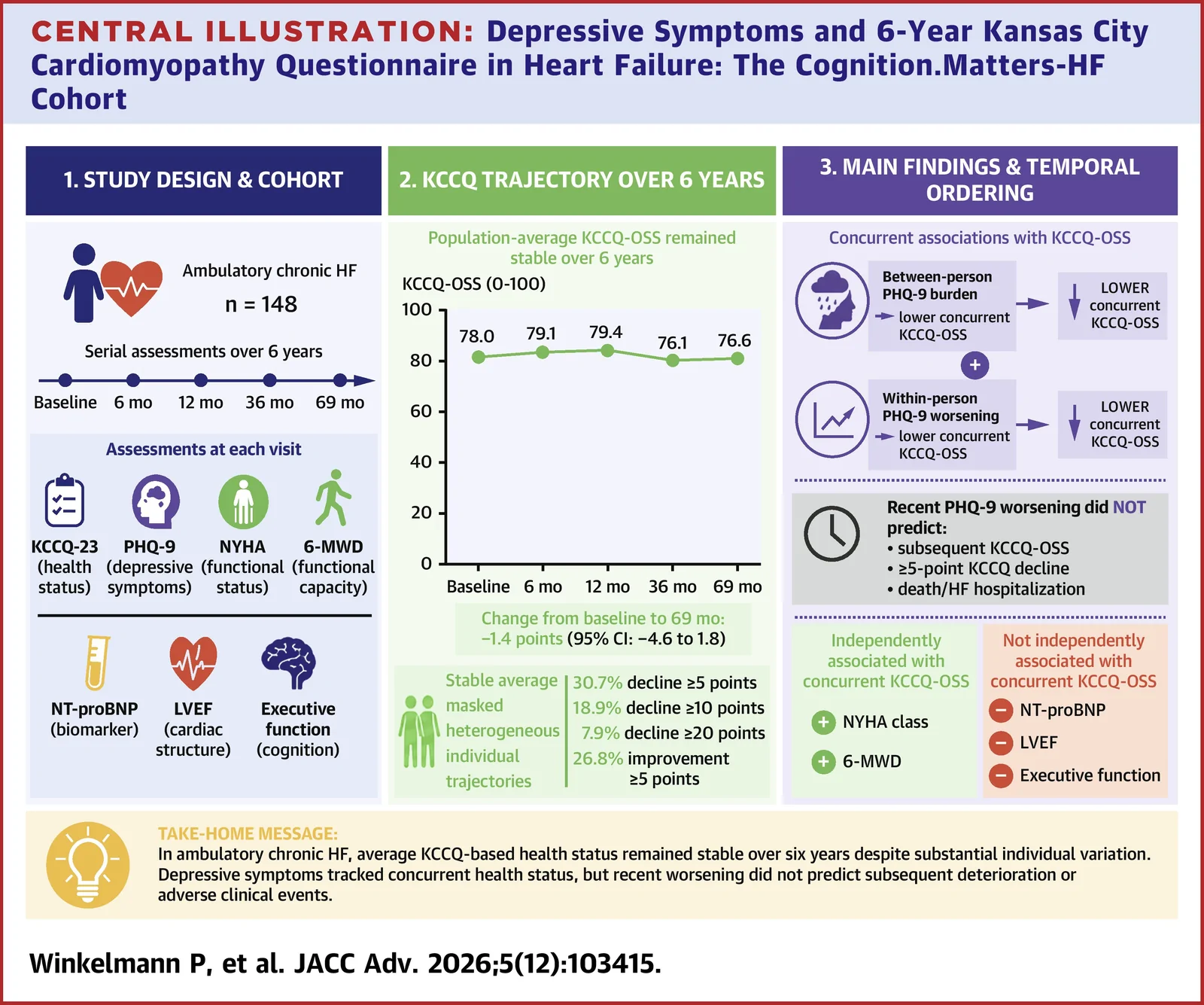
Depressive Symptoms and 6-Year Kansas City Cardiomyopathy Questionnaire in Heart Failure: The Cognition.Matters-HF Cohort In 148 ambulatory patients with chronic heart failure, population-average KCCQ-OSS remained stable over 69 months despite substantial individual variation. Higher persistent depressive symptom burden and visit-specific PHQ-9 worsening were independently associated with lower concurrent KCCQ-OSS. However, recent PHQ-9 worsening did not predict subsequent KCCQ-OSS, a clinically meaningful KCCQ decline, or subsequent all-cause death/HF hospitalization. NYHA functional class and 6-MWD, but not NT-proBNP, LVEF, or executive function, were independently associated with concurrent KCCQ-OSS. 6-MWD = 6-min walking distance; HF= heart failure; KCCQ-23 = 23-item Kansas City Cardiomyopathy Questionnaire; KCCQ-OSS = Kansas City Cardiomyopathy Questionnaire Overall Summary Score; LVEF = left ventricular ejection fraction; NT-proBNP = N-terminal pro–B-type natriuretic peptide; PHQ-9 = Patient Health Questionnaire-9.

### Persistent and visit-specific depressive symptom burden

The strong association between depressive symptoms and HF-specific health status in our cohort confirms rather than establishes this relationship. In REVIVAL, depression was strongly associated with lower KCCQ-OSS after accounting for other comorbidities,^3^ and Balata et al^4^ recently reported an inverse association between longitudinal changes in depressive symptoms and changes in KCCQ over 1 year. Our findings should therefore be viewed as an incremental extension of this literature.

The contribution of the present analysis is the separation of this established relationship into persistent between-person and visit-specific within-person components over nearly 6 years. Patients with higher average PHQ-9 burden had consistently lower KCCQ-OSS, whereas individual patients also reported lower KCCQ-OSS at visits when depressive symptoms were worse than their own usual level. This decomposition refines the longitudinal characterization of the depression–health-status relationship. However, because PHQ-9 and KCCQ were assessed contemporaneously at each visit, these findings alone do not demonstrate temporal precedence or causality, nor do they establish that the 2 components should lead to different clinical management strategies.

Our findings also fit with emerging HF literature demonstrating that depressive symptom burden is dynamic over time and that depressive symptom trajectories may carry prognostic information beyond a single baseline assessment.^18^

### Depression treatment and HF-specific health status

The strong association between depressive symptoms and KCCQ-OSS should not be interpreted to mean that treating depression alone will necessarily normalize HF-specific health status. Depression-targeted interventions in HF have shown that depressive symptoms can improve without parallel clinically meaningful improvements in KCCQ, suggesting partial decoupling between mood and HF-specific health status or the need for combined approaches. For example, collaborative care improved depressive symptoms but did not significantly improve KCCQ at 6 months.^5^ Pharmacological therapy in patients with HF and diagnosed depression did not improve clinical outcomes and did not clearly improve depression severity in MOOD-HF.^6^ More recently, a comparative effectiveness trial showed large reductions in depressive symptoms with both psychotherapy and antidepressants, with only small differences in general physical health status and health care use.^19^ Together with our findings, these data support embedding depression treatment within broader symptom-focused HF care, including functional rehabilitation, optimization of congestion management, and targeted supportive or palliative strategies, rather than expecting depression treatment alone to normalize KCCQ trajectories.

### Lack of predictors for the KCCQ-OSS trajectory

No covariate robustly predicted annual change in KCCQ-OSS. Thus, the present data do not identify clinical or psychological determinants of progressive long-term KCCQ deterioration. This should be interpreted in the context of the stable population-average trajectory, substantial individual heterogeneity, and limited power to detect differential slopes given long-term attrition. Importantly, the absence of slope predictors does not contradict the contemporaneous association between within-person PHQ-9 deviations and KCCQ-OSS level; these represent distinct longitudinal questions.

### Functional capacity, executive function, and patient-reported health status

Executive function was not independently associated with KCCQ-OSS after accounting for depressive symptoms and clinical status. In stable, ambulatory chronic HF, disease-specific health status may therefore be driven more directly by affective symptom burden and functional limitation than by higher-order cognitive control capacities. Alternatively, executive dysfunction may influence health status indirectly through self-care, adherence, physical activity, or participation, pathways that may not be fully captured by KCCQ-OSS. This interpretation is consistent with evidence from vascular cognitive impairment and stroke populations showing that cognitive impairment, including executive deficits, primarily affects activity and participation outcomes.^20^, ^21^

In contrast, NYHA functional class and 6-MWD were associated with KCCQ-OSS level, consistent with KCCQ reflecting patient-experienced functional limitation. Comparable studies demonstrate that KCCQ meaningfully discriminates risk strata and correlates with subsequent events, reinforcing its role as a patient-centered endpoint complementary to conventional measures.^22^ The lack of independent associations for NT-proBNP and LVEF further highlights that patient-reported benefit is not reducible to traditional physiological markers. For example, in AFFIRM-AHF, intravenous ferric carboxymaltose produced statistically significant and clinically relevant improvements in KCCQ-12 health status during follow-up.^23^

### Temporal ordering and clinical implications

The prospective analyses materially qualify the interpretation of the strong contemporaneous PHQ-9–KCCQ association. Although patients reported worse KCCQ-OSS at visits when depressive symptoms were worse than their own usual level, recent PHQ-9 worsening did not predict KCCQ-OSS at the subsequent visit, clinically meaningful subsequent KCCQ deterioration, or subsequent death or HF hospitalization. Thus, the within-person association appears primarily to reflect contemporaneous covariation in depressive symptoms and perceived HF health status rather than evidence that worsening depressive symptoms constitute an independent early marker of subsequent clinical deterioration.

Importantly, the contemporaneous association remained strong when depressive symptoms were restricted to the PHQ-2, which excludes the somatic PHQ-9 items most likely to overlap with HF symptoms. This suggests that the observed association is not solely explained by shared somatic symptom content, although shared patient-reported measurement and bidirectional effects cannot be excluded.

These findings do not demonstrate that repeated PHQ-9 assessment improves risk stratification or that PHQ-9 changes should trigger a specific HF management strategy. Rather, they refine the established relationship between depressive symptoms and HF-specific health status by distinguishing persistent and visit-specific contemporaneous components from prospective prediction. Whether structured clinical pathways combining repeated assessment of mood and HF-specific health status with actionable interventions improve patient-centered or clinical outcomes requires prospective interventional evaluation.

### Strengths and limitations

Key strengths include repeated standardized assessments over 6 years, use of linear mixed-effects modeling to leverage all available observations, decomposition of depressive symptoms into between- and within-person components, and multiple sensitivity analyses to evaluate robustness against informative dropout, including a joint longitudinal-survival model specifically accounting for dropout due to death.

Limitations primarily relate to generalizability, sample size, and attrition. The cohort was relatively small, recruited from a single center, and preselected for clinical stability and absence of overt neurological or psychiatric disease at baseline. This preselection may also have reduced the range of HF severity and symptom burden, thereby limiting the ability to identify determinants of progressive health-status deterioration. Applicability to broader HF populations with advanced HF, severe depression, stroke, or dementia may therefore be limited. Long-term attrition was substantial: of the 148 participants enrolled at baseline, 67 attended the 69-month visit, 29 had died, and 52 had dropped out or did not respond; KCCQ-OSS was available in 60 participants at 69 months. Although the complete-follow-up, MNAR delta-adjustment, and joint longitudinal-survival sensitivity analyses yielded broadly consistent results, these approaches cannot fully exclude residual selection toward healthier survivors. The modest cohort size and decreasing number of observations over time also limited statistical power, particularly for detecting predictor-by-time interactions and differential long-term trajectories. The joint longitudinal-survival model was used to address potential informative dropout due to death and should not be interpreted as a prognostic survival analysis of KCCQ-OSS. The additional prospective analyses were exploratory and based on available complete observations; their precision was limited by the modest number of longitudinal transitions and subsequent clinical events. Accordingly, the absence of statistically significant prospective associations should not be interpreted as excluding small effects. Finally, observational associations cannot establish causality; depressive symptoms may both influence and be influenced by perceived HF burden. Furthermore, women were under-represented in this cohort, limiting the ability to assess sex-specific associations or generalize findings equally across sexes. Gender identity was not assessed, reflecting the design and documentation standards of the original cohort.

## Conclusions

Population-average KCCQ-based health status remained stable over 6 years in ambulatory chronic HF despite substantial individual variation. Persistent and visit-specific depressive symptom burden were strongly associated with concurrent KCCQ-OSS, an association that persisted when analyses were restricted to core affective depressive symptoms, but recent worsening in depressive symptoms did not predict subsequent health-status deterioration or adverse clinical events. These findings refine the established longitudinal relationship between depressive symptoms and HF-specific health status without establishing incremental risk stratification or a specific management strategy.

Perspectives **COMPETENCY IN MEDICAL KNOWLEDGE:** In ambulatory patients with chronic HF, population-average disease-specific health status remained largely stable over 6 years despite substantial individual variation.

**COMPETENCY IN PATIENT CARE AND PROCEDURAL SKILLS:** Persistent and visit-specific depressive symptom burden were strongly associated with concurrent HF-specific health status, but recent worsening in depressive symptoms did not independently predict subsequent KCCQ deterioration or adverse clinical events.

**COMPETENCY IN INTERPERSONAL AND COMMUNICATION SKILLS:** Patient-reported depressive symptoms and HF-specific health status provide complementary information about the patient’s current perceived health burden, but the present data do not establish that serial PHQ-9 changes improve prospective risk stratification.

**TRANSLATIONAL OUTLOOK:** Future studies should determine whether structured clinical pathways linking repeated assessment of depressive symptoms and HF-specific health status to actionable interventions improve patient-centered or clinical outcomes.

## Funding support and author disclosures

This work was supported by public funding from the Federal Ministry of Education and Research, Bonn, Germany (01ES0816, 01ES01901, 01ES01902, 01EO1004, and 01EO1504), and the German Research Foundation, Bonn, Germany (391580509, 413657723, and 453989101). The authors have reported that they have no relationships relevant to the contents of this paper to disclose.
